# Sport Biomechanics Applications Using Inertial, Force, and EMG Sensors: A Literature Overview

**DOI:** 10.1155/2020/2041549

**Published:** 2020-06-23

**Authors:** Juri Taborri, Justin Keogh, Anton Kos, Alessandro Santuz, Anton Umek, Caryn Urbanczyk, Eline van der Kruk, Stefano Rossi

**Affiliations:** ^1^Department of Economics, Engineering, Society and Business Organization, University of Tuscia, Viterbo, Italy; ^2^Faculty of Health Science and Medicine, Bond University, Gold Coast, Queensland, Australia; ^3^Sports Performance Research Centre New Zealand, AUT University, Auckland, New Zealand; ^4^Cluster for Health Improvement, Faculty of Science, Health, Education and Engineering, University of the Sunshine Coast, Australia; ^5^Kasturba Medical College, Mangalore, Manipal Academy of Higher Education, Manipal, Karnataka, India; ^6^Faculty of Electrical Engineering, University of Ljubljana, Ljubljana, Slovenia; ^7^Atlantic Mobility Action Project, Brain Repair Centre, Department of Medical Neuroscience, Dalhousie University, Halifax, Nova Scotia, Canada; ^8^Department of Training and Movement Sciences, Humboldt-Universität zu Berlin, Berlin, Germany; ^9^Berlin School of Movement Science, Humboldt-Universität zu Berlin, Berlin, Germany; ^10^Department of Bioengineering, Imperial College London, London, UK

## Abstract

In the last few decades, a number of technological developments have advanced the spread of wearable sensors for the assessment of human motion. These sensors have been also developed to assess athletes' performance, providing useful guidelines for coaching, as well as for injury prevention. The data from these sensors provides key performance outcomes as well as more detailed kinematic, kinetic, and electromyographic data that provides insight into how the performance was obtained. From this perspective, inertial sensors, force sensors, and electromyography appear to be the most appropriate wearable sensors to use. Several studies were conducted to verify the feasibility of using wearable sensors for sport applications by using both commercially available and customized sensors. The present study seeks to provide an overview of sport biomechanics applications found from recent literature using wearable sensors, highlighting some information related to the used sensors and analysis methods. From the literature review results, it appears that inertial sensors are the most widespread sensors for assessing athletes' performance; however, there still exist applications for force sensors and electromyography in this context. The main sport assessed in the studies was running, even though the range of sports examined was quite high. The provided overview can be useful for researchers, athletes, and coaches to understand the technologies currently available for sport performance assessment.

## 1. Introduction

Recent statistics showed that about 50% of the European population performs a sport activity at least once a week starting from 15 years old [1]. It is well known that sports, or physical activities more generally, have a positive impact on quality of life. Several studies demonstrated the benefits in terms of life satisfaction, health, well-being, and educational and social participation [2, 3]. In addition, perhaps due to the growing number of people who compete in a wide variety of sports and recreational levels, the elite level requirements are constantly increasing. Recent technological developments have contributed to these increasing competitive levels, with these devices used to monitor sport training and competition performance, especially from a sport biomechanics perspective. Sport biomechanics represents the science that provides quantitative (and sometimes qualitative) assessments of sport performance; in particular, the kinematics and kinetics of sport movements [4]. Measuring and characterizing human movements during sporting activities are nowadays a crucial aspect for coaching programs in order to assess athletes' performance, to improve technique, and to prevent injuries [5–7]. In the past, 3D video analysis through optoelectronic systems represented the most widespread approach to analyse athlete behaviour during training or competition. Unfortunately, the 3D optoelectronic-based methodologies still have several limitations for widespread use in sport, such as difficulties in analysing human movement in outdoor environments, the time spent and the skills needed for the subjects' sensorization and the limited calibration volume in which the analyses can be performed [8]. The intrinsic limitations of using reflective markers, i.e. indoor analysis and competences required for the sensorization, have been overcome by markerless systems or specific processing systems, such as OpenSim or the use of artificial intelligence algorithms—for example, the concurrent neural network [9, 10]. Nowadays, sport biomechanics is, generally, performed by using wearable sensors that allow ensuring noninvasive data acquisition during the execution of movements [11]. Furthermore, wearable sensors allow the sporting activity to be performed in the natural environment, overcoming the environment limitation of laboratory testing, such as the use of the optoelectronic 3D system that is still considered the gold standard for movement analysis [11, 12]. Among others, inertial sensors [7, 13–49] force sensors [43, 50–70], and electromyography probes [71–137] are widely used for objectively and unobtrusively quantifying kinematics, kinetics, and muscle activity during sporting activities. One promising direction in wearable sensor use is real-time biofeedback systems [138] that can offer concurrent augmented feedback information to athletes and/or coaches [7, 139–142].

Although several systematic reviews already available in literature demonstrated the reliability, validity, and utility of inertial sensors for sport applications [8, 143, 144], an overview on specific applications that can be implemented by analysing kinematics, kinetics, muscle activity, and physiological parameters through wearable sensors is missing. From this perspective, we aimed to provide an overview on applications of sport biomechanics that require the use of wearable sensors, not only the inertial ones.

## 2. Materials and Methods

### 2.1. Search Strategy

Scopus, Web of Science, and PubMed databases were used to perform the literature review. Only studies that used wearable sensors for sport applications were considered; in particular, three categories were selected before the literature review: inertial sensors, force sensors, and electromyographic units. The start and the end of the literature review were July 2019 and November 2019, respectively. The following base keywords were used for the search: *sports*, *wearable sensors*, *wearable devices*, *biomechanics*, and *wireless.* More specifically, as regards inertial sensors, the following keywords were added: *IMU*, *inertial sensors*, *motion sensors*, and *wearable IMU*. Concerning the force measurements, *force* and *pressure* were used as additional keywords. As regards electromyography applications, these further keywords were used: *EMG*, *motor module*, *muscle coordination*, *muscle synergies*, *muscles*, *electromyography*, *patterned control*, *activation patterns*, *locomotor primitives*, and *modular organization*. In order to avoid bias in the search due to variations of root words, we also considered wildcard symbols, such as hyphens or inverted commas. The bibliography of the found studies was further checked in order to include relevant works accidentally omitted from the keyword-based research [11].

### 2.2. Inclusion Criteria

Studies were initially selected based on the relevance of the title and abstract. Thus, studies had to meet the following inclusion criteria: (i) only studies written in English were considered for the successive analysis, (ii) only studies published from 2010 onwards were included in order to avoid adding in the review outdated technologies, and (iii) conference proceedings were deleted if the same authors published also a journal paper regarding the same topic.

### 2.3. Data Extraction

Only studies that passed all the previous inclusion criteria were downloaded and managed through the Mendeley Desktop system. Since the review aimed at providing an overview of several wearable sensors used for sports, the studies were firstly categorized based on the type of wearable sensors used. The following information were gathered from each paper: (i) the aims, (ii) the examined sports, (iii) the kind of participants (e.g., inexperienced, recreational, and elite), (iv) the experimental setup, (v) how data was processed and analysed, and (vi) the results and conclusions. Studies that did not involve human subjects were automatically excluded.

### 2.4. Quality Assessment

The quality of each study was assessed in terms of internal, statistical, and external validity using the reported questionnaire [145]. All the authors were asked to answer an 18-item checklist, which is an optimization of similar ones used for reviews [146–150]. In particular, the checklist (Table 1) allowed us to assess information on internal (question numbers 1, 3, 4, 6, 7, 9, 12, 13, and 14), statistical (question numbers 15, 16, 17, and 18), and external (question numbers 2, 3, 5, 6, 8, 10, and 11) validity. The authors assigned a positive (one point) or negative (zero points) to each questionnaire item, and the final score was calculated by summing the assigned points. A study was considered as “high-quality” if it reached a score equal or greater than 11 (~61% of the maximum) in the evaluation of the majority of authors [147, 149]. Among the articles identified as “high-quality,” the authors selected a subset of papers that would be more fully examined in Results and Discussions. This selection was performed by considering only the studies that achieved a quality score of at least 15, in order to include studies in which the risk of bias was low.

For the sake of clarity, A.K. and A.U. performed the review of the inertial sensors, C.U. and E.K. took care of the force sensors, A.S. and J.T. performed the review of the EMG sensors, and J.T., J.K., and S.R. supervised the data quality assessment in order to avoid bias.

## 3. Results and Discussions

### 3.1. Inertial Sensors

The use of inertial sensors and wearable sensor devices in sports has boomed over the last decade. This is demonstrated by a simple search on Scopus using the keywords “sports” and “inertial sensors” that identified a total of 37 articles published in January to May 2020, a value that is identical to the number of articles found using the same search terms over the period 2004-2009. Modern inertial sensors are miniature low-power chips integrated into wearable sensor devices or smart equipment. Today's inertial sensors predominantly fall into the group of microelectromechanical systems (MEMS) that are portable, miniature, lightweight, inexpensive, and low power and generally include any combination of accelerometer, gyroscope, and magnetometer.

Inertial sensors are used for the measurement of static and dynamic states of the athlete's body. In the static state, some of the most important parameters are spatial position, orientation, posture, angles between body parts, etc. In the dynamic state, additional important parameters may include displacement, trajectory, velocity, linear acceleration, jerk (change of acceleration), angular velocity, angular acceleration, etc. While linear acceleration (accelerometer), angular velocity (gyroscope), and orientation (magnetometer) can all be measured directly, all other kinematic parameters must be derived from one or more measured quantities. For example, the velocity of a body is calculated by integrating its acceleration over time and its rotation angle is calculated by integrating its angular velocity over time. The measured and the derived results can be affected by inaccuracies of MEMS sensors. The discussion of this topic is not in the scope of this paper, but some useful guidelines on the proper use of MEMS inertial sensors can be found in [151–153].

**Figure 1:**
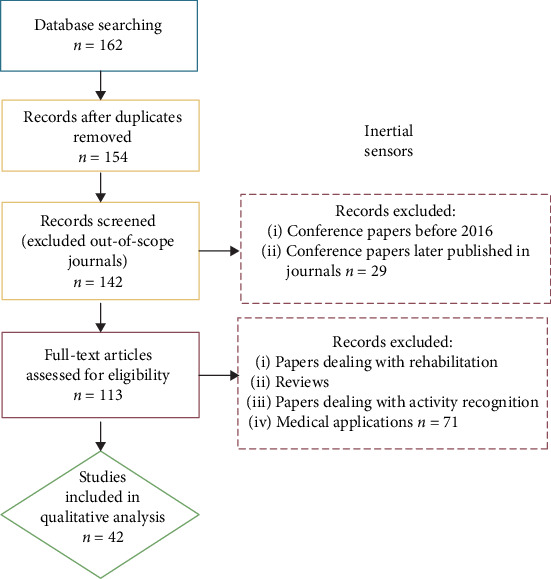
Selection process of papers focused on inertial sensors. Blue block represents the identification step, yellow blocks the screening step, red blocks the eligibility step, and green block the inclusion step.

The number of papers dealing with the use of inertial sensors in sport is far too great to process; a simple search in the SCOPUS database alone yielded over 1700 such papers. We have narrowed it down, as described in the search strategy in Section 2. The initial search, using the defined search terms, yielded 162 papers. After the author, duplicate, and language checks, 154 papers remained. After removing the older conference papers and conference papers that were later published in a journal, we have read the abstract of the remaining 113 papers. We have then excluded all review papers and articles concerning inertial, force, and EMG sensors, human activity detection, and detection of human states. From the remaining 64 papers, we excluded all general and non-sport-specific papers, which left 42 papers for thorough reading and analysis. The selection process is shown in Figure 1.

After analysing the chosen papers, we describe the use of inertial sensors based on the sport activity. More specifically, Table 2 shows the distribution of the included studies based on the specific sport.

Wearable sensor devices with integrated inertial sensors can be used for measuring and evaluating practically any activity in sport. Due to a large number of possible activities, we discuss the use of inertial sensors on a few groups of examples related to different sports.

Very frequent use of inertial sensors for various purposes was found in *walking and running actions*. The cyclic nature of such movements allows the use of a wide number of analysis techniques for the extraction of kinematic parameters or other results of interest. Analysing walking was perhaps the least difficult task within this group of actions, and there were numerous studies in this area. Flores-Morales et al. [21] used a mobile sensor device with six inertial sensors attached to the lower extremities of subjects and analysed the acquired data with the OpenSim system, which is open-source software, to create and analyse dynamic simulations of movement. An interesting approach, using the autocorrelation function for the assessment of regularity of cyclic human movements, including gait, was presented in [22]. A more energetic version of walking gait is Nordic walking. Nordic walking has been derived from snow skiing, whereby the individual uses handheld poles using a coordination pattern similar to cross-country skiing that requires substantially more upper body muscular involvement than typical walking movements. Derungs et al. [23] used 14 IMUs and regression methods for the estimation of acquired skills and detection of potential coordination mistakes in Nordic walking. The next step is using inertial sensors occurring in *running actions*. Since running is a more dynamic form of gait than walking, the requirements for sensors are higher. The determination of the foot strike pattern was the main idea in [24]. The authors used accelerometers and gyroscopes to calculate the stride length and determine the landing strategies at three running speeds. Similarly, Zrenner and colleagues [25] compared different statistical, DSP (Digital Signal Processing), and deep learning algorithms used for calculating the velocity and stride length in running using IMUs. Muniz-Pardos et al. [26] aimed to evaluate the running economy and foot mechanics in elite runners, which were determined through the use of an inertial sensor worn on the foot of the runners. The most dynamic action in this gait group is *sprint*. An accelerometer positioned on the sprinters' waist was used in [27] for the assessment of sprint based on the regression machine learning method. Mertens et al. [28] employed sophisticated validation methods including laser pistols and real-time kinematic GPS systems for the measurement of the sprint velocity using only one IMU with an integrated accelerometer and gyroscope.

Another group of activities, where inertial sensors can be extremely beneficial, are *racket and bat sports*. A typical use of IMU in such actions is on the hand/wrist/arm of the athlete or integrated into the equipment. Wang and colleagues [29] devised an Internet of Things (IoT) platform for use in racket sports. They placed an IMU on the wrist of the athlete and processed the acquired data through the machine learning methods. They performed skill assessments that sought to differentiate between professional, subelite, and amateur *badminton* players just from their stroke performance. Similar approaches and methods were used in [30], where authors devised a system with three IMUs attached to the hand, wrist, and elbow of the athlete. The system employed deep learning methods for providing useful information to coaches in *table tennis* practice. Among racket sports, tennis seems to be the most popular for using inertial sensor systems. Yang et al. [31] used two IMU devices attached to the wrist and the knee of the athlete to evaluate the *tennis* serve performance through the support vector machine method. Very similar goals were presented in [32], where authors used three gyroscope sensors attached to the hand, upper arm, and chest of the athlete. They used DSP, statistical, and simulation methods for the assessment of the first serve skill in tennis. Stroke detection and classification were the main result of the paper [33]. The authors used a wrist-worn IMU and decision tree machine learning methods to detect and classify three most common tennis strokes: forehand, backhand, and serve with 98.1% accuracy. Human movement coordination assessment with the use of three IMUs at the hip, wrist, and chest of the athlete was presented in [34]. The authors evaluated *the baseball swing* movement based on the template matching method and give feedback to the athletes and coaches. Capturing fast athletic biomechanics was the core of the work presented in [35], whereby IMUs were positioned on the chest, upper arm, wrist, hand, and waist to acquire high dynamic movements with the combination of the multirange accelerometers and gyroscopes. For the high-dynamic movements, the accelerometers and gyroscopes with ±200 *g*_0_ and ±20000°/s were used, respectively. For the low-dynamic movements, the accelerometers and gyroscopes with ±16 *g*_0_ and ±1000°/s were used, respectively. The result of their work was a wearable dual-range sensor platform that enabled an investigation of high-level, very wide dynamic-range biomechanical parameters describing the baseball swing.

*Team sports* are also very interesting for research but may get complex because of the interactions, unpredictability, and nonuniformity of athlete actions. Studies of the sport activities in group sports were mostly limited to isolated specific movements of one athlete. Wang and colleagues [36] used one IMU at the wrist of the athlete to assess the skill level of a *volleyball* spiker. The recorded data was classified into three levels: elite, subelite, and amateur volleyball players with 94% accuracy. *Basketball* was also popular with researchers; Ma et al. [37] and Meng et al. [38] used a wrist-worn sensor to recognize and classify basketball movements using support vector machine classification methods. In [37], nine kinds of basic basketball movements, such as stand, walk, run, jump, in situ dribble, dribble while walking, dribble while running, set shot, and jump shot, were recognized. Shankar et al. [39] described the mobile system that enabled remote monitoring of shooting form of a basketball player. One IMU was attached to the wrist of the athlete that collects shooting data, and a heuristic classification method was used to estimate the shooting performance according to the efficiency calculated as the ratio of the shots made to the total number of shots taken by the player in a given range of flick velocities and loading angles. Results show that the player's shooting action improved and became more consistent within his preferred trajectory over the course of 3 weeks of training with the device. With wider use of machine learning algorithms in team sports, new possibilities of detecting and identifying group events at training and matches have become possible. Chambers and colleagues [154, 155] have designed algorithms based on the random forest for automatic detection of tackle, ruck, and scrum events in rugby union. During the match play, they achieved the classification accuracy of 79.4% (ruck), 81.0% (tackle), and 93.6% (scrum).

The next group of activities is sports where athletes move themselves with the aid of different equipment. We chose to report a few studies within the group of *skiing sports*, where athletes use different forms of skis to perform the desired action. The authors of [40] used deep learning techniques to analyse the data from 17 IMU devices attached to the *cross-country* skier. The result was the classification of the eight classical and skating style cross-country techniques based on the data from 5 most relevant IMUs with the accuracy of 87.2% and 95.1% for the flat and natural course, respectively. *Ski jumping is* an interesting winter sport discipline from the perspective of measurement of kinetic and kinematic parameters. Bessone et al. [42] used 11 IMUs to determine the possible correlation between kinematics and kinetics during landing. Analysis methods included DSP, statistics, and iSEN system software. The results can be used during daily training, giving specific feedback on the ways of reducing the vertical ground reaction force at landing. The most complex and dynamic of the studied winter skiing sports is *alpine skiing*. Analysis of motion of the lower extremities during the carving technique is performed in [43], where authors used 17 IMUs placed over the skier's body. The acquired data was processed and analysed by DSP algorithms, motion analysis capture system, and multiscale computer simulation. Fasel et al. [44] used 6 IMUs to capture the three-dimensional body and centre of mass kinematics of an alpine skier, with this IMU data augmented by a differential GPS system giving the location of the skier's COM on the skiing slope. Yu and colleagues [45] studied the potential of using IMU sensors for performance analysis of alpine skiers. They used 16 IMUs to find the best location of the sensor. The findings, based on the statistical analyses and the hierarchical clustering methods, suggested that the best location was the pelvis, as this may quite accurately reflect the total body's COM position.

From a number of implementation perspectives, the most challenging activities for the application of inertial units are *water sports*. For example, wearable sensor devices must be waterproof; therefore, their design and construction are more challenging and expensive. Also, radio signals do not penetrate water well; therefore, wireless communication with a sensor device underwater is practically impossible. Wang et al. [46] used one 9 degree-of-freedom IMU to capture the posture of the human lumbar spine during swimming. In order to quantify the spinal motion during swimming, they used an orientation estimation algorithm and a human biomechanical model. Their sensor system collected the data offline and transferred it wirelessly to the PC after swimming, when the swimmer gets out of the water. Lecoutere and Puers [47] used a low-power wireless sensor network and wearable sensor device attached to the head of the swimmer to track elite swimmers in real-time. Their wearable sensor device uses gyroscope and accelerometer signals to calculate the most important swimming parameters locally and sends them to the PC at the times when the swimmer's head is out of the water. A similar approach was performed by Kos and Umek [7], where one IMU with an accelerometer and gyroscope was attached to the low back to acquire a number of the most relevant swimming parameters for all four swimming disciplines. Their sensor device recorded the swimming data offline and transfers them to the PC after swimming using a wired connection.

### 3.2. Force Measurement Devices

**Figure 2:**
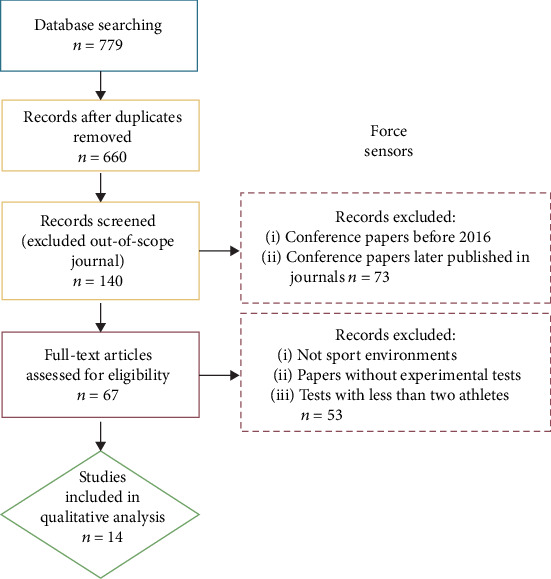
Selection process of papers focused on force sensors. Blue block represents the identification step, yellow blocks the screening step, red blocks the eligibility step, and green block the inclusion step.

Forces acting on (or generated by) an athlete can provide valuable insight into their likely performance and injury risk. Variables based on stand-alone force measurements include centre of pressure (CoP) [50], direction of the force as a proxy measure of efficiency [51], and impact forces [52]. Combined with kinematic measurements, force data have been used to estimate mechanical power [53], joint kinetics [43, 54], and muscle forces [55]. Analysing kinetics in the laboratory is mostly done with force plates which are typically embedded in the floor. This setup is however static and often does not allow the kinetics to be assessed during the actual sporting activity due to the inability to instrument the playing surface with a sufficient number of force platforms. Measurements of forces in sport applications therefore require wearable force measurement devices or specifically instrumented surfaces such as starting blocks in swimming or athletics which can only provide data on the race start. The selection process for paper inclusion is reported in Figure 2.

Table 3 shows the distribution of the included studies based on the specific sport.

The literature on wearable force devices can roughly be divided into studies that use commercially available (off-the-shelf) pressure sensors and studies that use custom-built devices. Articles were selected in which the wearable systems were used in a setting that evaluated the biomechanics of athletes.

#### 3.2.1. Commercially Available Systems

Pressure sensors are commercially available measurement devices that can be directly applied in an experimental sport setup. A list of commercially available devices used in literature is reported in Table 4. Pressure sensors convert physical pressure into an electric current or voltage. To estimate force, the pressure is multiplied by the area over which that pressure is applied. The number of sensors (how much target area is covered by the sensors) is therefore an important determinant for the accuracy of the system. Apart from the number of sensors, accuracy of the individual pressure sensors is determined by resolution, hysteresis, repeatability, and linearity. In the case of insoles, fit inside the shoes is important. In skating and skiing, shoes are often tight fitting, custom made, and thermoformed, which requires insoles that are customizable, for example, with the option to cut them in the right shape. A limitation of pressure sensors is that they measure the pressure only in one direction. A major advantage of these portable sensors is that they can be used in many different environments and sports.

As regards the evaluation of the CoP, Buckeridge et al. (2015) used insoles (Pedar X, Novel, Munich, Germany) to determine the CoP and foot pressure in elite and recreational ice hockey players in acceleration and steady-state forward skating. Although the plantar forces measured by the insoles were not different between elite and recreational athletes, a finding consistent with speed skating studies [58, 59], the CoP was different between the level of athletes. Elite players had their CoP more to the forefoot compared to recreational players during steady-state skating [50]. Although in this study only forward skating was considered, this measurement setup with insoles is applicable for the assessment of other locomotive activities performed in ice hockey games.

As regards the evaluation of the joint kinetics, two studies in literature used pressure insoles (Pedar X, Novel, Munich, Germany) in combination with an MVN motion capture suit comprising 17 IMUs (Xsens, Enschede, The Netherlands) to analyse joint kinetics in skiing [43] and short-track speed skating [54]. Combining kinematics from the Xsens suit with the measured plantar forces from the pressure insoles, an inverse dynamics analysis was performed to obtain the joint kinetics (intersegmental rigid body kinematics). Lee et al. [43] showed that hip, knee, and ankle joint forces and moments, calculated based on a standard inverse dynamics analysis using the motion capture data and ground reaction force, for middle-turn were higher compared to those for short-turn in ski carving. Purevsuren et al. [54] concluded that short-trackers have high internal rotational moments when the knee is flexed. This conclusion might however not be valid since pressure insoles can only estimate the force component normal to the plantar surface and hence moment (free moment) and force components parallel to the plantar surface. Instead, forces in the horizontal plane are significant in short-track speed skating [60]. Moreover, only straight forward skating was incorporated in the analysis, whereas most of the time skaters are either entering, exiting, or inside a curve [60]. The researchers may have been limited in measuring this part of the rink due to the high centrifugal forces that disturb the IMU-based measurement systems [61]. Apart from inaccuracies in force measurement, IMU-based (joint) kinematics are more inaccurate than optoelectronic measurement systems, which are currently regarded as “*gold standard*” [12]. In speed skating, a sensitivity analysis of joint power estimation using an inverse dynamics model of a speed skater showed that the model was most sensitive to the COM position of the trunk and the lean and steer angle of the skates (rotating the locally measured forces into a global frame). A 5° inaccuracy of the skate's steer angle, which is likely to occur in IMU-based systems [61, 156], resulted in approximatively 9.5% maximum error in the joint power estimations compared to optoelectronic systems. It should also be acknowledged that the inverse dynamics approach, even in laboratory situations, has some limitations relating to a variety of assumptions (e.g., use of rigid body segments) that may result in errors of approximately 10–20%. The reliability and value of this combination of systems for sport performance enhancement may still be somewhat limited.

As regards contact forces, commercially available pressure sensing components have also been integrated into custom arrangements for force sensing in specific applications. Jennings et al. [57] created a linear array of individual force-resistive pressure sensors (Flexiforce, A201-25, Tekscan) mounted to the head of a field hockey stick to measure the forces and CoP between the ball and stick during a goal shooting skill called a drag flick. The study determined that force and location of the ball along the stick were important for controlling the trajectory of the ball during the drag flick, and the simple sensing array was able to distinguish the skill level among athletes based on consistency of the force patterns and decreasing overall contact time [57].

An alternative to the insole systems discussed above, shoes or footplates may be instrumented with a custom arrangement of sensors. Sturm et al. [56, 62] mounted a rectangular array of individual force-resistive pressure sensors (Flexiforce, A201-100, Tekscan) to measure foot force transfer from kayaking athletes into the boat. In kayak racing, foot force has an important effect on the whole-body rhythm/movement pattern used to “kick” the boat forward, and this is evident in the alternating push-pull force displayed within each foot and also by the 180° phase difference in force timing between left and right feet.

#### 3.2.2. Custom Systems

While pressure sensors are valuable for assessing normal force distributions, they cannot measure out-of-plane forces. Several studies have therefore constructed custom measurement devices to examine forces in three dimensions. Constructions usually incorporate commercially available load cells or strain gauges.

#### 3.2.3. Instrumented Impact Plates

In the classic sense of a wearable device, Saponara [52] developed a wireless instrumented plate designed to be worn within the athlete clothing for measuring contact force during martial art sparring. The system comprised a matrix of strain gauge sensors to sense deformation of a thin aluminium plate under load from a kick or punch. Depending on the specific sport usage, several plates could be worn on the chest, shoulders, legs, and arms and linked by a microcontroller (HX711, Sparkfun) and Bluetooth to a single data acquisition program. They tested their system with a broad range of karate athletes, measuring contact time and force of strikes. The authors defined two performance metrics (kick-strength-to-weight ratio (KSWR) and punch-strength-to-weight ratio (PSWR)) and gave feedback to athletes using a grading scale from poor to excellent. The authors found a correlation between system measurements, effectiveness of leg/arm movement, and athlete skill level (i.e., years of experience). The authors suggest that the coordinative skill of the more experienced athletes allows them to more efficiently utilise the kinetic link principle, thereby ensuring a greater transfer force through the kinetic chain to the feet and hands when performing kicks and punches [52].

#### 3.2.4. Instrumented Speed Skates

Although skates had been instrumented prior to 2010 using strain gauges [63–66], van der Kruk et al. built the first wireless instrumented speed skates for short-track (fixed blade) [60] and long-track (klapskates) [58] speed skating. The instrumentation is located in the bridge (klapskates) and cups (fixed blade) of the skates, each consisting of a sandwich construction that clasps piezoelectric three-component force sensors (Kistler 9602, Kistler Group, Winterthur, Switzerland). This allows measurement of the lateral and normal forces on the skates. The output of the sensor is logged on a SD card and sent over Bluetooth via a data logger that is attached to the skates. The instrumented short-track skates were used in the routine training of Olympic athletes. Within this homogenous group, higher-ranked male skaters tended to have a CoP more to the rear of the blade and lower lateral forces for several phases (curve, leaving the curve, and entering the straight) of skating [60]. Females showed a trend towards applying higher body weight normalised lateral forces than males, while skating at lower velocities, which is suggested to reflect body weight, muscular strength, and/or motor control differences between females and males while skating on the same blades [60]. Since lateral forces and the CoP determine the heading (steering) of the skate, this seems to be an important performance indicator that can be tracked with these wearable force platforms. Limitations for the current design of these skates are the additional weight, and, in the case of the instrumented short-track skate, the slight height difference may alter the feel and performance of the typical movement.

#### 3.2.5. Instrumented Saddle

Analogous to [43, 54], Walker et al. [67] combined 5 IMUs (Xsens, Enschede, The Netherlands) to record gross body movement with axial load cells mounted within the stirrups of a horse racing saddle, underneath the jockey's feet. They compared the kinematics and kinetics of jockeys while galloping on a riding simulator with actual horse racing. The authors found that stirrup force amplitudes on real horses were more than twice those recorded on the simulator and were asymmetric, with higher peak forces applied to the stirrup opposite the horse's lead leg while the jockey's pelvis displaced laterally away from the lead leg, suggesting that jockeys use their legs and hips to isolate their centre of mass and dampen the effects of the horse's movement [67].

#### 3.2.6. Instrumented Baseball

Often in ball sports, a strictly wearable device does not provide all the information of interest. This is especially true in ball throwing sports, like baseball, where a pitcher's choice of pitch type dictates finger position around the ball and effects the forces imparted by the fingers onto the baseball and the resulting trajectory of a pitch [51]. Kinoshita et al. (2017) embedded a triaxial load cell (USL06-H5-500N-C, Tec Gihan Co., Kyoto, Japan) in a Japanese league regulation baseball and recorded timing and amplitude of finger forces during fastball pitches. The embedded transducer was wired by a quick release mechanism to a data logger worn on the athlete's wrist, such that the connection would detach when the ball left the pitcher's hand [51]. The authors found that all fingers generated a peak force amplitude 37-43 ms before ball release, while the index and middle fingers displayed bimodal force patterns with an additional peak 6-7 ms before ball release. Peak ball reaction force exceeded 80% of maximum finger strength, and there was a linear relationship of peak force with ball velocity. Because of space limitations within the ball, they were unable to record all finger forces simultaneously. Instead, the hand was carefully repositioned between trials such that the appropriate finger of interest would overlay the force sensor. This does however introduce the possibility of crosstalk, which the authors acknowledge as a study limitation.

#### 3.2.7. Instrumented Paddles

In kayaking, the athlete's paddle acts effectively as an extension of their arm for force generation. Providing feedback to athletes and coaches about the magnitude and shape of paddle force-time curves at different paces can have implications for performance and training. Two research groups [56, 68] independently developed paddle-mounted force systems where the shaft was instrumented with two sensor nodes, each comprising strain gauges (HBM, Darmstadt, Germany) in a Wheatstone bridge configuration. The FPaddle system developed by Gomes et al. [68] used 2 strain gauges directly bonded to the carbon fibre composite paddle with nodes located 80 cm from each blade tip, while Sturm et al. [56, 62] created a self-contained system with 4 strain gauges bonded to a cantilever beam and held in place on the paddle by a clamp mechanism. Gomes et al. [68] showed that on-water force-time profiles change in magnitude and shape with the increased stroke rate, with higher mean paddle force more strongly correlated with increased kayak velocity than peak paddle force. The authors also reported an efficiency metric—the ratio of mean force to peak force—which reflected shape changes in the force-time profiles and related this to stroke impulse (i.e., the integral of the force-time profile).

#### 3.2.8. Future Implications

In addition to limitations of data transfer bandwidth and sampling rate, studies utilising customised external equipment still indicate that the ecological validity of these studies is still not perfect. Specifically, athletes were still aware of the additional weight in the equipment and concerned that this may lead to performance reductions, meaning the implementation of these tools in competition scenarios may not be currently advisable. Jennings et al. [57] noted that additional instrumentation mounted to the field hockey stick may effect ball contact and trajectory. Kinoshita et al. [51] quantified the decline in ball velocity (11% of self-reported max speed) as a function of the added load cell weight, which as a result could not be used in a regulation match. The authors also discussed concerns about impact forces of the ball against a bat or catcher's mitt and the potential for fatally compromising the instrumentation. Lastly, as with all on-water, ice, or snow-oriented sensor packages, waterproofing electronics is necessary but can be expensive and heavy, and if the sealing was to become compromised, also potentially hazardous [56, 60, 68].

### 3.3. Surface Electromyography

**Figure 3:**
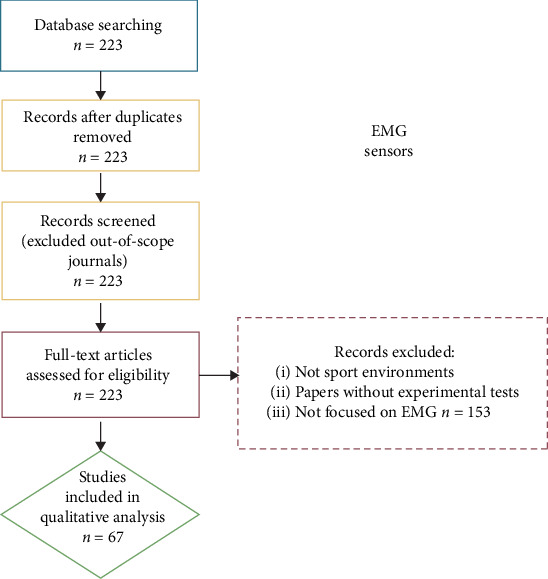
Selection process of papers focused on EMG sensors. Blue block represents the identification step, yellow blocks the screening step, red blocks the eligibility step, and green block the inclusion step.

The applications of surface electromyography (sEMG) in sport science have become increasingly common and diversified in the last decade [157]. Possibly also thanks to the advent of wireless systems, sEMG is nowadays largely used not only as a descriptive tool but also in quantitative studies as well. Bipolar (i.e., employing a set of two electrodes) setups are popular in sport science to record noninvasively the summation of action potentials over the skin, giving as output an analogue signal that describes the electric potential difference (voltage) detected between the two electrodes [157]. Through specific postprocessing procedures, such as rectification and filtering of the signal, researchers can use multimuscle sEMG recordings to describe and/or quantify coordinated activations orchestrated by the central nervous system (CNS) to produce and control movement [71–137]. Of the 67 studies considered in this section, around half followed classic approaches for the analysis of sEMG [71–74, 76–115], leading to the computation of amplitude, timing, and frequency parameters. Another 31 adopted the muscle synergy framework to analyse the data [75, 116–136]. The selection process for the paper inclusion is shown in Figure 3.

#### 3.3.1. Amplitude, Timing, and Frequency Content of sEMG

The most common approach to the analysis of sEMG signals is the assessment of the maximum or mean amplitude of the envelope, with or without normalisation to the maximum voluntary contraction [71–74, 76–87, 89–95, 97–106]. The analysis of timing is also common in sport science, with usual approaches ranging from the detection of the onset and offset of sEMG activity and global and local maxima detection to examination of the entire time course using statistical parametric mapping [72–74, 76–79, 81, 83, 89–93, 96, 100, 102]. Other advanced approaches include the analysis of the signal's frequency content, especially for fatigue estimation [79, 106], classification of sEMG patterns through *k*-means clustering or support vector machines [82, 88], and nonlinear analysis of the signals using the Lyapunov exponents [102]. The majority of the studies included the recordings of less than nine muscles [72, 73, 77–85, 87, 91–95, 98–106, 158], while only a few considered a number between nine and 16 [74, 76, 86, 88, 90, 96] or bigger than 16 [71, 97]. Most of the studies considered muscles of the lower limb [71–73, 77, 78, 81, 83–85, 89, 90, 92, 93, 95, 96, 98, 104, 106, 115], with the remaining focussing on the trunk and/or upper limb [74, 76, 80, 82, 87, 94, 100, 102, 103, 106] or both the upper and lower body [79, 86, 88, 91, 97, 99, 101]. Bilateral recordings (involving the left and right hand side of the same muscles) were less common [71, 74, 77, 82, 86, 88, 90, 97, 98, 101] than ipsilateral [72, 73, 76, 78–81, 83–85, 87, 89, 91–96, 99, 100].

#### 3.3.2. Muscle Synergies

The concept of muscle synergies is based on the fact that the CNS must constantly deal with an overabundant number of degrees of freedom [159]. Based on the seminal work of Bernstein [159], Bizzi and colleagues proposed that the CNS might simplify the production and control of movement by activating muscles in groups rather than individually, in common patterns called synergies [160]. Even though a direct experimental proof for this theory is currently missing, muscle synergies are increasingly being used in sport science to either speculate on the physiological meaning of coordinated muscle activation patterns or present multimuscle sEMG recordings in a compact way. Muscle synergies are in fact obtained by the factorisation of sEMG signals, a numerical procedure that allows for the reduction of dimensionality of big data sets through decomposition techniques such as nonnegative matrix factorisation (NMF), principal component analysis (PCA), independent component analysis, and factor analysis [116, 127, 161]. All of these methods reduce sEMG time series to a set of motor modules (time-invariant muscle weights), which describe the relative contribution of single muscles within a specific synergy and a set of motor primitives (time-dependent coefficients), representing the common activation patterns. Studies on the reliability of muscle synergy extraction in relation to sport activities are scarce but nevertheless present in the considered literature [108, 116, 127, 128]. The most common family of algorithms used to reduce the dimensionality of the data was NMF [107, 108, 110–114, 116–125, 127–133, 135–137], with a few studies also using PCA to extract synergistic muscle activations [75, 109, 126, 134]. The total number of muscle activities recorded varied heavily across the considered studies. We found a range in the number of muscles recorded across these studies, including one to eight [108, 109, 124, 126, 131, 133], between nine and 16 [75, 107, 110–114, 116, 119, 121–123, 125, 128, 130, 132, 134–137], and between 16 and 25 muscles [117, 118, 120, 127, 129]. Bilateral recordings were less common [107, 108, 117, 119, 129] than ipsilateral [75, 109-114, 116, 118, 120–128, , 128, 130–137]. Most of the studies considered muscles of the lower limb [75, 108, 109, 112–114, 116, 119, 123, 124, 126, 130, 133, 136], even though almost as many muscles are included from the trunk and/or upper limb as well [110, 111, 117, 118, 120, 125, 127–129, 131, 132, 134]. Only three studies focused exclusively on the upper body [107, 135, 137].

#### 3.3.3. Sport Application with EMG

The studies considered analysed a rather broad spectrum of sport activities (Table 5). The most represented activity was running, although this was assessed in a variety of conditions including overground or on treadmill, shod or barefoot, level or incline, at different speeds, and on even or uneven surfaces [83, 84, 89, 90, 92, 93, 95, 96, 111, 113, 116, 118, 123, 126, 127, 129, 130, 133, 136]. A lot of attention was also given to resistance training or weightlifting [78, 80, 81, 85, 86, 94, 97, 102, 103, 105, 108, 125, 128] and to cycling or handcycling [71–76, 104, 109, 112, 114, 119, 124]. Swimming is also getting increasing interest in recent years [82, 87, 91, 106, 131] as are ball sports such as softball, baseball, or cricket [98–101]. We found that less attention was given to sports such as rowing [120, 134], golf [88, 117], rugby or American football [77, 137], cross-country skiing [79], gymnastics [135], ice hockey [132], pole vaulting [107], and skateboarding [115]. Among those studies, it is interesting to notice how the use of sEMG to quantify injury risk or recovery is still very limited [81, 88, 90, 104].

There is, however, a new branch of sport science that employs perturbations as either the pivotal component of training interventions or the mean to investigate the responses of the CNS in balance-challenging conditions. Perturbation has to be intended as a change of movement, as reported in the Oxford dictionary. Perturbations can be used to uncover motor control processes that under unperturbed circumstances would not be available for observation [162]. Of the six studies that dealt with perturbations, four have been published after March 2017, indicating an increasing interest in the topic by the sport science community [97, 102, 103, 110, 121, 136]. A brief review of those six works is presented in the following lines. Kohler and colleagues calculated the average root mean square (RMS) of the sEMG signal recorded from eight ipsilateral muscles of the upper limb and trunk while lifting stable (barbell) and unstable (dumbbell) loads on stable (bench) and unstable (Swiss ball) surfaces in a seated overhead shoulder press [103]. They found the highest RMS values of the *triceps brachii* sEMG activity when lifting the stable load on a stable surface, while the lowest values were associated with lifting of unstable loads on an unstable surface. Based on those observations, the authors concluded that training interventions centred on lifting overhead unstable loads and/or surfaces might not benefit the development of core muscle strength. A similar conclusion was also drawn in another study that reported no significant correlation between three measures of core muscle strength and the difference in dumbbell overhead shoulder press strength when assessed on a stable bench compared to an unstable Swiss ball [163]. In a similar fashion, Nairn and colleagues analysed the amplitude of the linear envelope of the sEMG signals recorded from 12 bilateral muscles of the trunk and lower limbs during a squat exercise while lifting stable (Olympic bar) and unstable (water-filled cylinder, only on a stable surface) loads on stable (solid ground) and unstable (BOSU ball) surfaces [97]. The authors found that unstable loads on stable surfaces reduced the activation of the *erector spinae* but increased the activation of the *abdominal external oblique* compared to stable loads. However, lifting stable loads on unstable surfaces increased the activation of more distal muscles, such as *gastrocnemius medialis*, *biceps femoris*, and *vastus medialis*. The conclusion from this study was that altering the stability of the support surface and/or the stability of the load to be lifted can have differing effects on the muscle activity of the agonist compared to stabiliser muscles. Lawrence and colleagues set out to investigate the stability of sEMG signals recorded from eight bilateral muscles of the trunk and upper limbs during bench press involving stable (standard barbell) and unstable (flexible barbell with loads suspended by elastic bands) loads [102]. The authors calculated the Lyapunov exponents of the sEMG signals but did not specify if they computed the short- or long-term exponents. They concluded that unstable loads were managed by reducing the instability of sEMG signals (i.e., lower Lyapunov exponents). de Brito Silva et al. extracted synergies from the muscle activity of 12 lower limb muscles recorded during single-leg landing from a lateral jump on a stable surface [121]. Then, they proceeded to train the participants on an unstable surface (wobble board) three times a week for four weeks and assessed the effects of training on muscle synergies. The authors reported a modified modular organisation of muscle activation patterns after wobble board training, but no changes in the number of muscle synergies. Specifically, the landing strategy switched to a separation of the relative contribution of the plantarflexors (*gastrocnemius medialis* and *gastrocnemius lateralis*) from the dorsiflexors and mediolateral stabilisers (*tibialis anterior* and *peroneus longus*, respectively). Moreover, the relative contribution of secondary muscles within each motor module decreased. The authors concluded that wobble board training modifies the modular organisation of landing redistributing the relative contribution of muscle groups in a function-specific way. Oliveira and colleagues analysed the influence of perturbations (translation of support surface) on the modular organisation of direction changes during running [110]. The setup consisted in recording the sEMG activity of 16 ipsilateral muscles of the lower limb and trunk during 90° side-step cutting manoeuvres while running with and without translation of the solid support surface at contact. The results showed no differences in the number of muscle synergies and minor effects of perturbations on motor modules, while motor primitives underwent stronger modifications. The authors concluded that the timing properties of motor primitives were likely influenced by sensory input and descending command integration. Santuz et al. investigated the effects of terrain morphology on the modular organisation of running [136]. The experimental setup consisted of a standard and an uneven-surface treadmill, on which the participants ran while the sEMG activity of 13 ipsilateral muscles of the lower limb was recorded. Similar to the studies of de Brito Silva et al. and Oliveira et al., the authors found that the number of muscle synergies was not affected by the uneven surface. Moreover, the changes in the motor modules due to the challenging terrain were subtle. The changes in the motor primitives, however, were visible in the weight acceptance and propulsion synergies. Specifically, the primitives of those two synergies were significantly wider in the uneven surface as compared to the even surface condition. The authors concluded that the widening might be a strategy adopted by the CNS to make chronologically adjacent primitives overlap. This would increase the robustness (i.e., ability to cope with errors) of the motor output when locomotion is challenged by external perturbations.

Taken together, these results show that perturbations can be used to study those motor control processes that under unperturbed circumstances would not be available for observation. This allows for a better understanding of a complex system such as the CNS not only from a basic research point of view but also from an applied research perspective as well. The studies mentioned above highlight the specific role of the perturbation type and location in modulating the activity of determined muscle groups [97, 103, 121] and how activation patterns are modulated by the CNS in challenging settings [102, 110, 136]. Perturbation-based studies and training interventions are becoming ever more popular, and researchers as well as coaches will likely benefit, in the near future, from a wider body of literature.

## 4. Conclusions

The assessment of motor performance in sports is becoming more and more important due to the high level of competition and financial rewards among athletes. Wearable sensors have the potential to provide key data relating to training and competitive performance. Among other sensor options, inertial sensors are the most widespread, even though force measurement systems and electromyography allow further information on the kinetics, and associated muscle activity levels can provide additional insight into the motor behaviours of athletes. From the analyses, it should be also underlined that some methodologies, for example, the computation of joint moments from the pressure insoles, need to be validated before they are more commonly used in the field of sport biomechanics to ensure that such data is methodologically solid, meets the metrological requirements (accuracy, reliability, and repeatability), and is meaningful for the field of sport biomechanics. The outcomes of this literature review provide sport scientists (including biomechanists), coaches, and athletes an overview on sport biomechanics applications that required the use of wearable sensors.

## Figures and Tables

**Table 1:** Criteria for quality assessment of the internal validity (IV), statistical validity (SV), and external validity (EV).

Criteria		Assessment property
*Aim of the work*		
(1)	Description of a specific, clearly stated purpose	IV
(2)	The research question is scientifically relevant	EV
*Inclusion criteria (selection bias)*		
(3)	Description of inclusion and exclusion criteria	IV-EV
(4)	Inclusion and exclusion criteria are the same for all tested groups	IV
(5)	Inclusion and exclusion criteria reflect the general population	EV
*Data collection (performance bias)*		
(6)	Data collection is clearly described and reliable	IV-EV
(7)	Same data collection method used for all the athletes	IV
(8)	The used setup is wearable	EV
Data loss (attrition bias)		
(9)	Different data loss between groups	IV
(10)	Data loss < 20%	EV
*Outcome (detection bias)*		
(11)	Outcomes allow tangible application	EV
(12)	Outcomes are the same for all the athletes	IV
*Data presentation*		
(13)	Frequencies of most important outcome measures	IV
(14)	Presentation of the data is sufficient to assess the adequacy of the analysis	IV
*Statistical approach*		
(15)	Appropriate statistical analysis techniques	SV
(16)	Clearly state the statistical test used	SV
(17)	State and reference the analytical software used	SV
(18)	At least 10 subjects	SV

**Table 2:** References of inertial sensor measurement based on different analysed sports.

Sport/function	Number of studies	References
Gait	2	[21, 22]
Nordic walking	1	[23]
Running	3	[24–26]
Sprint	2	[27, 28]
Badminton	1	[29]
Table tennis	1	[30]
Tennis	3	[31–33]
Baseball	2	[34, 35]
Basketball	3	[37–39]
Volleyball	1	[36]
Rugby union	2	[154, 155]
Cross-country skiing	1	[40]
Roller skiing	1	[41]
Ski jumping	1	[42]
Alpine skiing	3	[43–45]
Swimming	2	[46, 47]

**Table 3:** References of force measurement based on different analysed sports.

Sport	Number of studies	References
Ice hockey	1	[50]
Baseball	1	[51]
Karate	1	[52]
Skiing	2	[43, 70]
Speed skating	3	[54, 60, 61]
Field hockey	1	[57]
Kayaking	3	[56, 62, 68]
Horse riding	1	[67]
Golf	1	[69]

**Table 4:** Variety of commercially available pressure insoles used in sport studies. Characteristics are based on data sheets provided by the manufacturers and via direct contact with the manufacturers where necessary.

Company	Product name	Technology	Measuring range (kPa)	Number of sensors (#)	Sam. Freq (Hz)	Recording time (h)	Thickness (mm)	Weight (g)	Wireless	Costs (€)
3L Labs	Footlogger	—	nk	8	500	24	3	Unknown		<1000
Moticon	OpenGo	Capacitive	500	16	100	0.5-16	3.2	116 (incl. battery)	X	1k-5k
Novel	Pedar X	Capacitive	600/1200	99-256	80-200	4.5	1.9	400 (incl. data box)		>10k
Orpyx	Kinetyx	Resistive	nk	37	256	12	nk	Unknown	X	1k-5k
SPI	Tactilus HP	Resistive	200	128	300	nk	1.3	Unknown		5k-10k
Tekscan	F-scan	Resistive	517/862	954	500	2	0.4	332 (incl. data box)		>10k

nk = unknown.

**Table 5:** References of electromyography applications based on different analysed sports.

Sport	Number of studies	References
Running	19	[83, 84, 89, 90, 92, 93, 95, 96, 111, 113, 116, 118, 123, 126, 127, 129, 130, 133, 136]
Resistance training or weightlifting	13	[78, 80, 81, 85, 86, 94, 97, 102, 103, 105, 108, 125, 128]
Cycling or handcycling	12	[71–76, 104, 109, 112, 114, 119, 124]
Swimming	5	[82, 87, 91, 106, 131]
Softball, baseball, or cricket	4	[98–101]
Rowing	2	[120, 134]
Golf	2	[88, 117]
Rugby or American football	2	[77, 137]
Cross-country skiing	1	[79]
Gymnastics	1	[135]
Ice hockey	1	[132]
Pole vaulting	1	[107]
Skateboarding	1	[115]
